# Algal Bioremediation of Waste Waters from Land-Based Aquaculture Using *Ulva*: Selecting Target Species and Strains

**DOI:** 10.1371/journal.pone.0077344

**Published:** 2013-10-15

**Authors:** Rebecca J. Lawton, Leonardo Mata, Rocky de Nys, Nicholas A. Paul

**Affiliations:** School of Marine and Tropical Biology, James Cook University, Townsville, Queensland, Australia; Stazione Zoologica, Italy

## Abstract

The optimised reduction of dissolved nutrient loads in aquaculture effluents through bioremediation requires selection of appropriate algal species and strains. The objective of the current study was to identify target species and strains from the macroalgal genus *Ulva* for bioremediation of land-based aquaculture facilities in Eastern Australia. We surveyed land-based aquaculture facilities and natural coastal environments across three geographic locations in Eastern Australia to determine which species of *Ulva* occur naturally in this region and conducted growth trials at three temperature treatments on a subset of samples from each location to determine whether local strains had superior performance under local environmental conditions. DNA barcoding using the markers ITS and *tufA* identified six species of *Ulva*, with *U. ohnoi* being the most common blade species and *U. sp.* 3 the most common filamentous species. Both species occurred at multiple land-based aquaculture facilities in Townsville and Brisbane and multiple strains of each species grew well in culture. Specific growth rates of *U. ohnoi* and *U. sp. 3* were high (over 9% and 15% day^−1^ respectively) across temperature treatments. Within species, strains of *U. ohnoi* had higher growth in temperatures corresponding to local conditions, suggesting that strains may be locally adapted. However, across all temperature treatments Townsville strains had the highest growth rates (11.2–20.4% day^−1^) and Sydney strains had the lowest growth rates (2.5–8.3% day^−1^). We also found significant differences in growth between strains of *U. ohnoi* collected from the same geographic location, highlighting the potential to isolate and cultivate fast growing strains. In contrast, there was no clearly identifiable competitive strain of filamentous *Ulva,* with multiple species and strains having variable performance. The fast growth rates and broad geographical distribution of *U. ohnoi* make this an ideal species to target for bioremediation activities at land-based aquaculture facilities in Eastern Australia.

## Introduction

Many intensive aquaculture industries generate nutrient-rich waste water streams which, if untreated, can cause eutrophication of coastal waters and negatively impact downstream biological communities [1], [2]. The use of live algae to remove excess dissolved nutrients from aquaculture effluents – algal bioremediation - is widely accepted as an efficient and cost effective waste water treatment method [3]–[7]. This is particularly the case for land-based aquaculture operations, where production is often limited by strict environmental regulations around water quality of point-source discharges [8], [9]. The application of algal bioremediation at these sites can reduce nutrient loads in effluents prior to discharge, thereby providing an opportunity to increase feed inputs and consequently farm productivity [10].

A significant reduction of nutrient loads in aquaculture effluents through algal bioremediation requires the selection of appropriate species. A range of characteristics should be considered when choosing species for bioremediation. It is essential for species to have high growth rates as this generally translates into high bioremediation capability [3], [7]. Species should also be able to grow across a wide range of conditions [11], with the aim of year round production in open culture systems. Additionally, species should occur locally and, if possible, have a broad geographic distribution [3], [7], [12]. This will minimise the risk of cultivated species invading natural ecosystems and impacting on native biodiversity. It may also identify target species within diverse genera that have more suitable traits for bioremediation, including growth and nutrient uptake [13], [14].

Selecting appropriate target species is therefore the first critical step in implementing an algal bioremediation programme. If algal bioremediation is to be used at multiple locations then the second critical step is to consider whether a single strain of the target species is used across all locations or local strains are isolated and used at each location. (We use the term strain to refer to individual samples or variants within a species, including ecotypes or genotypes). If there is a strong genotype × environment (G × E) interaction, local strains may possess traits that provide an advantage under local conditions leading to superior performance under these conditions compared to strains originating from other habitats [15]. Adaptation of populations to local environments has been demonstrated across a range of phototropic organisms including terrestrial plants [16], [17], aquatic plants [18] and algae [19]–[21]. Based on these results, we would expect local strains of algae to have higher growth, and therefore bioremediation capability, in their local habitats compared to non-local strains. However, experimental studies have not always found that local strains are superior under local environmental conditions [22]–[26]. Therefore, assessing the performance of strains sourced from multiple locations under a range of environmental conditions is important in the selection of target species for algal bioremediation.

The objective of the current study was to firstly identify target algal species, and secondly identify strains, for bioremediation of land-based aquaculture facilities in Eastern Australia. Specifically, we first wanted to identify target species with high growth rates and a broad distribution along the east Australian coastline. Such a species could then be used for algal bioremediation across multiple aquaculture facilities, resulting in a consistent supply of algal biomass for commercial applications and removing biosecurity concerns associated with the use of non-endemic species. Second, we wanted to determine whether local strains of the selected species had superior performance under local environmental conditions. Many marine algae species may be suitable for bioremediation; however, we focus here only on species from the genus *Ulva.* Species from this genus are ideal candidates for bioremediation of aquaculture effluents as they have high growth rates, a broad environmental tolerance, and relatively low susceptibility to epiphytism [9], [27]–[30]. In addition, species of *Ulva* can rapidly absorb and metabolise inorganic phosphorus and nitrogen – the two primary nutrients of concern in intensive aquaculture [27]–[30]. Furthermore, many *Ulva* species have a cosmopolitan distribution in Eastern Australia and are commonly found in coastal intertidal habitats [31], [32]. To achieve our aims, we surveyed coastal environments across three locations in Eastern Australia over 14° of latitude, including land-based aquaculture facilities, to determine which species of *Ulva* occur naturally in this region and in aquaculture bioremediation areas. We then maintained strains of algae collected from all three locations in laboratory cultures and conducted growth trials on a subset of strains which had survived in culture for more than three months. These trials tested the hypothesis that locally derived strains have higher growth rates under local conditions.

## Methods

### Sample Collection

Eighty-four strains of *Ulva* were collected from intertidal environments in three distinct regions – Townsville (19°S, 146°E), Brisbane (27°S, 153°E) and Sydney (33°S, 151°E) – along the east coast of Australia, and six land-based aquaculture facilities located in the Townsville and Brisbane regions. Permission was obtained from owners and local authorities where appropriate to collect *Ulva* from these sites. As strains were collected during the dry season period, salinity levels were constant (∼36^0^/_00_) between locations. Strains were transported in water taken at the collection site back to James Cook University, Townsville, where they were maintained in nutrient enriched autoclaved seawater (Guillards F/2 medium; 12.3 mg L^−1^ nitrogen, 1.12 mg L^−1^ phosphorus ) in a temperature and light controlled laboratory (12∶12 light: dark cycle, 49.2 µmol photons m^−2^ s^−1^, 23°C). These nutrient values are similar to those found in aquaculture facilities [8]. A small subsample of each specimen was dried and retained as a voucher. Forty strains which survived in culture for more than four weeks were identified to species level using DNA barcoding (Table 1; Table S1) due to the well established difficulties associated with identifying *Ulva* specimens to species using morphological and cytological characteristics [32]. DNA barcoding is an alternative approach to species identification for groups in which phenotypic plasticity is an issue, cryptic taxa are likely to exist, and morphological keys are ineffective for particular life stages or genders [33]. This approach compares short DNA sequences from a standardised region of the genome - the ‘barcode’ - to a library of reference sequences derived from individuals of known identity [33].

**Table 1:** Sample information.

				Accession number^2^	
Species^1^	Strain	Collection site	Morphology	ITS	*tufA*
*Ulva compressa*	BA2	Bare Island, NSW	Filament	KF195484	KF195520
*U. fasciata*	BI2	Bribie Island, QLD	Blade	KF195485	KF195521
U/I	BI4	Bribie Island, QLD	Blade	KF195486	KF195522
*U. sp. 3*	BI6	Bribie Island, QLD	Filament	KF195487	U/R
*U. ohnoi*	BI9	Bribie Island, QLD	Blade	KF195488	KF195523
*U. ohnoi*	BI13	Bribie Island, QLD	Blade	KF195489	KF195524
*U. sp. 3*	BI15	Bribie Island, QLD	Filament	KF195490	KF195525
*U. torta*	CL2	Clovelly, NSW	Filament	KF195491	KF195526
*U. fasciata*	CL7	Clovelly, NSW	Blade	U/R	KF195527
*U. australis*	CL8	Clovelly, NSW	Blade	KF195492	KF195528
*U. compressa*	CL9	Clovelly, NSW	Filament	KF195493	KF195529
*U. compressa*	CL10	Clovelly, NSW	Filament	KF195494	KF195530
*U. ohnoi*	C02	Coogee, NSW	Blade	KF195495	KF195531
*U. ohnoi*	GC1	Gold Coast Marine, QLD	Blade	KF195496	KF195532
*U. ohnoi*	GFB1	Good Fortune Bay Fisheries, QLD	Blade	KF195497	KF195533
*U. sp. 3*	GFB2	Good Fortune Bay Fisheries, QLD	Filament	KF195498	U/R
*U. ohnoi*	GFB5	Good Fortune Bay Fisheries, QLD	Blade	KF195499	KF195534
U/I	GFB6	Good Fortune Bay Fisheries, QLD	Filament	KF195500	KF195535
*U. ohnoi*	JCU1	James Cook University, QLD	Blade	KF195501	KF195536
U/I	JCU2	James Cook University, QLD	Filament	KF195502	KF195537
*U. sp. 3*	JCU3	James Cook University, QLD	Filament	KF195503	KF195538
*U. ohnoi*	KP1	Townsville, QLD	Blade	KF195504	KF195539
*U. ohnoi*	KP2	Townsville, QLD	Blade	KF195505	KF195540
*U. ohnoi*	KP3	Townsville, QLD	Blade	KF195506	KF195541
*U. ohnoi*	MA1	Malabar, NSW	Blade	KF195507	KF195542
*U. compressa*	MA6	Malabar, NSW	Filament	KF195508	KF195543
*U. fasciata*	MA9	Malabar, NSW	Blade	KF195509	KF195544
*U. fasciata*	MR3	Maroubra, NSW	Blade	KF195510	KF195545
*U. intestinalis*	MR4	Maroubra, NSW	Filament	KF195511	KF195546
*U. fasciata*	MR5	Maroubra, NSW	Blade	KF195512	KF195547
*U. sp. 3*	PR3	Pacific Reef Fisheries, QLD	Filament	KF195513	KF195548
*U. ohnoi*	PR4	Pacific Reef Fisheries, QLD	Blade	KF195514	KF195549
*U. ohnoi*	RC1	Redcliffe, QLD	Blade	KF195515	KF195550
*U. compressa*	RC3	Redcliffe, QLD	Filament	U/R	KF195551
*U. ohnoi*	RC6	Redcliffe, QLD	Blade	KF195516	KF195552
*U. sp. 3*	SA4	Australian Prawn Farms, QLD	Filament	KF195517	KF195553
U/I	SB1	Caloundra, QLD	Filament	U/R	KF195554
*U. sp. 3*	SB5	Caloundra, QLD	Filament	KF195518	KF195555
*U. sp. 3*	SB12	Caloundra, QLD	Filament	KF195519	KF195556
*U. sp. 3*	TV3	Townsville, QLD	Filament	U/R	KF195557

Collection information, mophology and GenBank accession numbers for ITS and *tufA* sequences for all study samples. See Table S1 for further details.

1 U/I – unidentifiable,

2 U/R – sequence unreadable.

### Species Identification

We used the DNA barcode markers *tufA* and ITS to identify strains of *Ulva*. The plastid elongation factor *tufA* has been used to discriminate amongst green algal species in a range of studies [31], [34] and this marker had high levels of discriminatory power between species in a recent evaluation of barcode markers for marine green macroalgae [35]. The internal transcribed spacer region of the ribosomal cistron (ITS) has been widely used in species-level phylogenetic studies of green algae [36]–[38], including several studies of *Ulva* species in Australia [32], [39].

A small piece of fresh tissue was isolated from each strain, rinsed in autoclaved seawater and scrapped to remove any epiphytes. Total DNA was extracted from this tissue using a Qiagen DNEasy Plant Mini Kit following the manufacturer’s instructions. The *tufA* region was amplified using the primers tufGF4 and tufAR [35]; the ITS region was amplified using the primers ITS1 [36] and G4 [40]. Polymerase chain reaction (PCR) amplifications were performed in 25 µL reaction mixture containing 1.5 U of MyTaq HS DNA polymerase (Bioline), 5×MyTaq reaction buffer, 0.4 µM of each primer and 0.5 µL of genomic DNA (25–30 ng). Amplifications were performed on a BioRad C1000 Thermal Cycler with a touchdown PCR cycling profile (cycling parameters: 5 min at 94°C, 30 cycles of 30 s denaturing at 95°C, 45 s annealing at 60°C with the annealing temperature decreasing by 0.5°C each cycle, 60 s extension at 72°C, and a final extension at 72°C for 5 min). PCR products were column purified using Sephadex G-25 resin and sequenced in both directions by the Australian Genome Research Facility (Brisbane, Australia). If sequences were unreadable or appeared to contain mixed template a second PCR attempt was performed and products resequenced. Sequences were edited using Sequencher v 4.5 (Gene Codes Corporation, Ann Arbor, MI, USA) and submitted to GenBank under the accession numbers given in Table 1.

Strains were identified based on their DNA sequences by constructing phylogenetic trees using sequences downloaded from Genbank. All publically available *tufA Ulva* sequences published as part of peer reviewed journal articles were downloaded. Due to the large number of ITS *Ulva* sequences in Genbank we downloaded all newly generated sequences from the recent phylogenetic studies of Australian *Ulva* by Kraft et al. [39] and Hawaiian *Ulva* by O’Kelly et al. [41] and all previously published ITS *Ulva* sequences used in these two studies for analysis. Duplicate sequences were removed from each dataset and then all remaining sequences were aligned with ours and trimmed to a standard length in MEGA 5.0 [42]. The *tufA* dataset included 78 sequences, 39 of which were retrieved from GenBank and the alignment consisted of 660 positions. The ITS dataset included 104 sequences, 68 of which were retrieved from GenBank and the alignment consisted of 595 positions. Maximum likelihood (ML) and neighbour joining (NJ) phylogenetic trees were constructed in MEGA using an *Ulvaria obscura* sequence (*tufA*: HQ610405; ITS: AY260571) as an outgroup. jModelTest 2.1 [43], [44] showed that the TIM3+G model of molecular evolution best fitted the *TufA* data and the TIM1+G model best fitted the ITS data. However, as these models were not available in MEGA we used the simple Kimura two-parameter model to estimate genetic distance [45] as this is the standard model of molecular evolution used in barcoding studies [46]. The reliability of tree topologies was estimated using bootstrapping (1,000 replicates). As both maximum likelihood and neighbour joining trees produced very similar outcomes we only present results based on the maximum likelihood tree.

### Growth Trials

To determine which naturally occurring species of *Ulva* would be suitable for targets for algal bioremediation, growth trials were conducted under three temperature treatments on a subset of samples from each location. Strains were categorised as having either a blade or filamentous morphology and three strains of each morphology from each location, where possible, which had survived in culture for more than three months were selected for these experiments. Only two strains with blade morphology from Sydney were used in the experiment as all other strains from this location did not survive in culture. The two species with blade morphology used in the experiment were *U. ohnoi* (Sydney, Brisbane, Townsville) and *U. fasciata* (Sydney). Notably, *U. ohnoi* was the only blade species to survive the initial three month period of growth across all locations, and subsequently, seven of the eight strains used in the growth trial are *U. ohnoi*. As multiple strains of filamentous *Ulva* survived in culture, strains from four species were used in the experiment – *U. compressa* (Sydney, Brisbane), *U. intestinalis* (Sydney), *U. sp. 3* (Brisbane, Townsville) and an unidentified species (strain JCU2, Townsville). Nine replicates of standardised size were isolated from each strain using a 6 mm diameter hole punch for blade morphologies and an 8 mm diameter hole punch for filamentous morphologies. Three replicates of each strain were then grown at each of three temperatures (17.5°C, 23°C, and 28.5°C) in culture cabinets with 12 hour light: 12 hour dark cycles and a light level of 49.2 µmol m^−2^ s^−1^ for 7 days. These temperatures were chosen to be representative of the minimum average monthly sea surface temperature in Sydney (17.5°C), the average monthly sea surface temperature in Brisbane (23°C), and the maximum average monthly sea surface temperature in Townsville (28.5°C) (http://www.metoc.gov.au/products/data/aussst.php). This range of temperatures also represents the lower range of Townsville (23°C), the upper range of Brisbane (28°C) and the upper range of Sydney (23°C).

Each individual replicate was maintained in a sterile 60 mm petri dish with nutrient enriched autoclaved seawater and photographed under a stereo dissecting microscope at the start and end of the 7 day period to determine the 2- dimensional surface area. Specific growth rates were calculated for each individual replicate of each strain using the equation SGR (% day^−1^) = Ln(B_f_/B_i_)/T*100, where *B_f_* and *B_i_* are the final and initial surface areas (mm^2^) and *T* is the number of days in culture. Growth rates for blades and filaments were analysed separately. Permutational analyses of variance (PERMANOVAs) were used to analyse the effect of collection location (Sydney, Brisbane and Townsville) and temperature on specific growth rate of blades and filaments. As seven of the eight blade strains included in the experiment were the species *U. ohnoi*, we also separately analysed the effect of temperature and strain on specific growth rates of this species. This analysis allowed us to statistically test whether there were differences in the growth rates of strains within a single species. All analyses were conducted in Primer v6 (Primer-E Ltd, UK) using Bray-Curtis dissimilarities on fourth root transformed data and 999 unrestricted permutations of raw data [47].

## Results

### Species Identification

**Figure 1:**
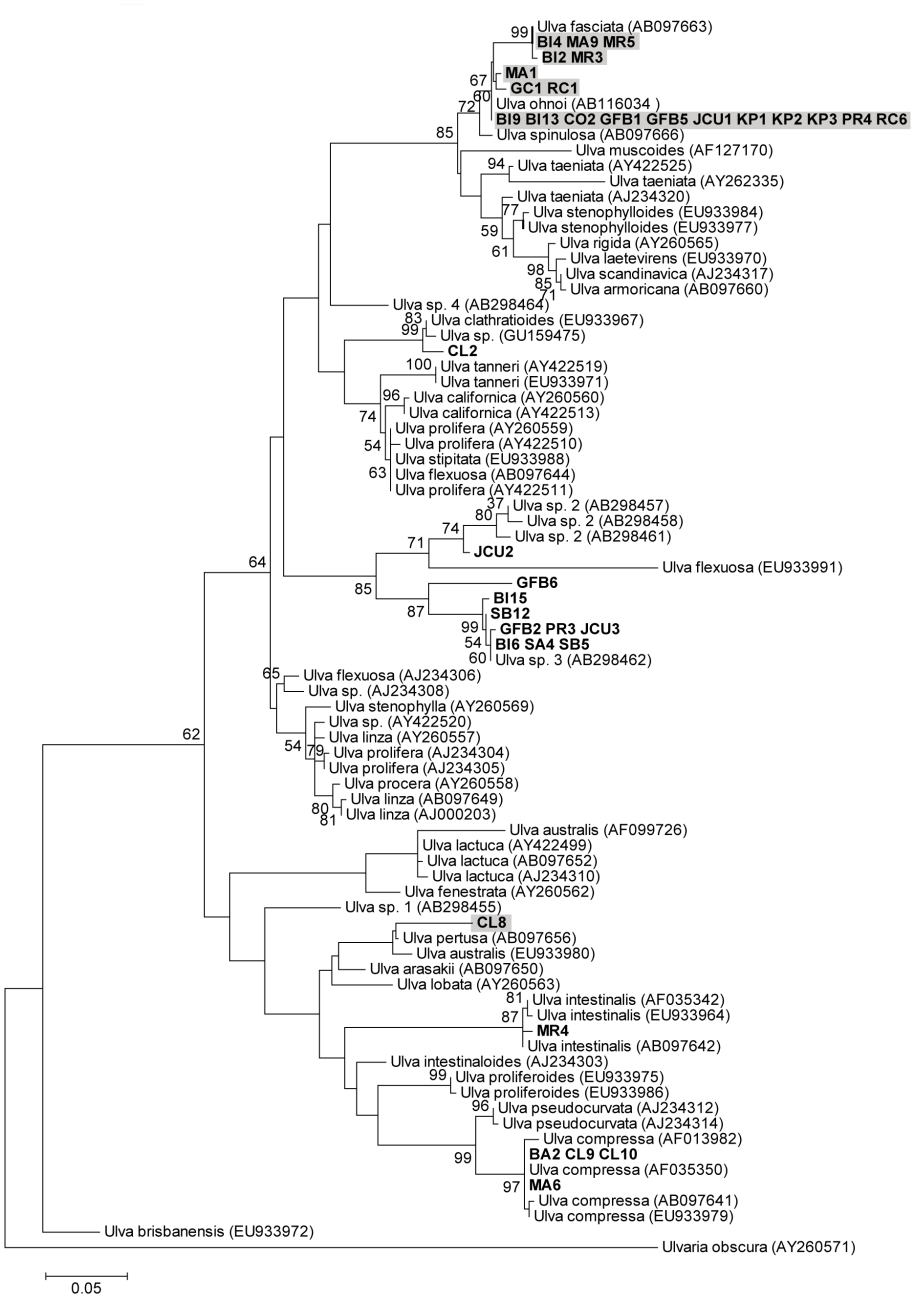
*Ulva* ITS phylogenetic tree. Maximum likelihood tree of *Ulva* internal transcribed spacer (ITS) sequence data (scale at bottom). Numbers near each node refer to bootstrap support values, nodes with <50% bootstrap support are not labelled. Samples collected in this study shown in bold. Shading indicates strains with blade morphologies. Numbers accompanying the species names are GenBank accession numbers for the sequences used in the analysis.

**Figure 2:**
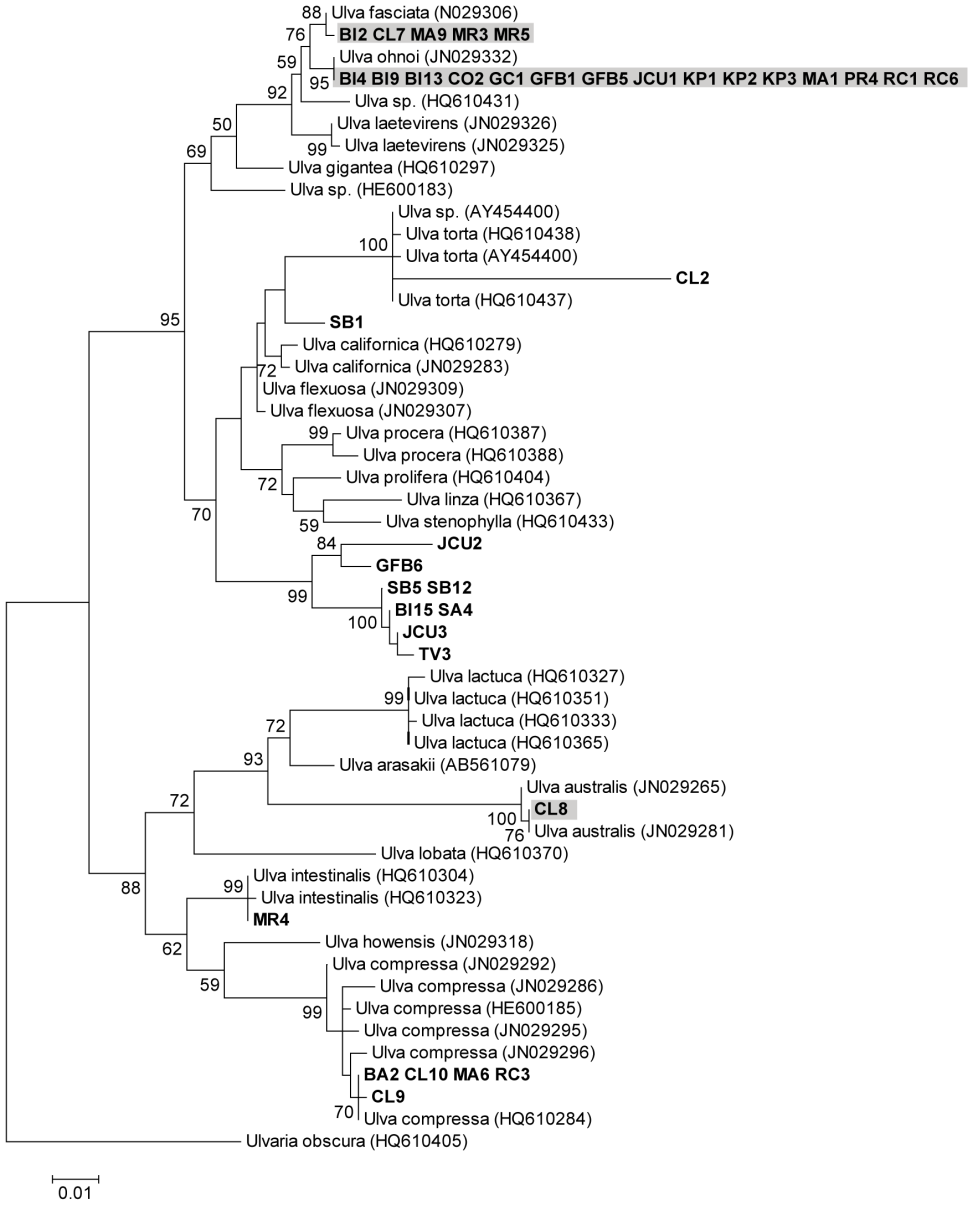
*Ulva tufA* phylogenetic tree. Maximum likelihood tree of *Ulva tufA* sequence data (scale at bottom). Numbers near each node refer to bootstrap support values, nodes with <50% bootstrap support are not labelled. Samples collected in this study shown in bold. Shading indicates strains with blade morphologies. Numbers accompanying the species names are GenBank accession numbers for the sequences used in the analysis.

Based on the *tufA* and ITS phylogenetic trees we were able to assign species names to 35 of our 40 strains (Table 1, Figs. 1 & 2). Twenty-one strains were assigned to the same species by both *tufA* and ITS sequence data (Table S1). Nineteen of these strains had identical ITS and *tufA* sequences to GenBank samples of *U. ohnoi* (BI9, BI13, CO2, GFB1, GFB5, JCU1, KP1, KP2, KP3, PR4, RC6), *U. fasciata* (BI2, MA9, MR3, MR5) and *U. compressa* (BA2, CL9, CL10, MA6). A further two strains had identical *tufA* or ITS sequences to GenBank samples of *U. australis* (CL8) and *U. intestinalis* (MR4), and their sequences for the remaining marker formed clades (99% bootstrap support for MR4) with GenBank samples of these species. GC1, MA1, RC1 were identified as *U. ohnoi* as they had identical *tufA* sequences to GenBank *U. ohnoi* samples and their ITS sequences fell within a supported clade (72% bootstrap support) that contained *U. ohnoi* and the closely related *U. fasciata*. Eight strains (BI6, BI15, GFB2, JCU3 PR3, SA4, SB5, SB12) had ITS sequences that formed a distinct, well supported (99% bootstrap support) clade with a GenBank sample from Japan identified as *U. sp. 3* [AB298458, 48]. The *tufA* sequences of BI6 and GFB2 were unreadable and the *tufA* sequences of the remaining six strains (BI15, JCU3, PR3, SA4, SB5, SB12) formed a distinct clade with strong support (100% bootstrap support) that did not include any GenBank samples with which they could be identified. *tufA* sequences are not available for the Japanese *U. sp. 3* sample. However, we have provisionally called these eight strains *U. sp. 3* due to their similar grouping in both the *tufA* and ITS phylogenies and the high support (99% bootstrap support) for these clades. Strain TV3 has also been provisionally called *U. sp. 3* as, although its ITS sequence was unreadable, the *tufA* sequence was identical to the six other strains provisionally named as *U. sp. 3.* ITS sequences were unreadable for CL7 and RC3, however, as their *tufA* sequences were identical to GenBank samples for *U. fasciata* and *U. compressa* respectively they have been assigned to these species. Species identification of BI4 was not possible as the *tufA* sequence for this strain was identical to *U. ohnoi*, while the ITS sequence was identical to *U. fasciata,* suggesting that it may be a hybrid of these two species. Similarly, species identification was not possible for CL2 as the ITS sequence for this strain formed a clade with *U. clathratioides* GenBank samples, while the *tufA* sequence formed a clade with *U. torta* Genbank samples. Species identification was also not possible for GFB6, JCU2 or SB1 - in both the ITS and *tufA* phylogenies, each of these strains formed a distinct clade that did not contain any other samples. In both the ITS and *tufA* phylogenies there was no clear grouping of blade or filamentous strains, with both types of morphologies occurring within multiple nodes of the trees.

**Figure 3:**
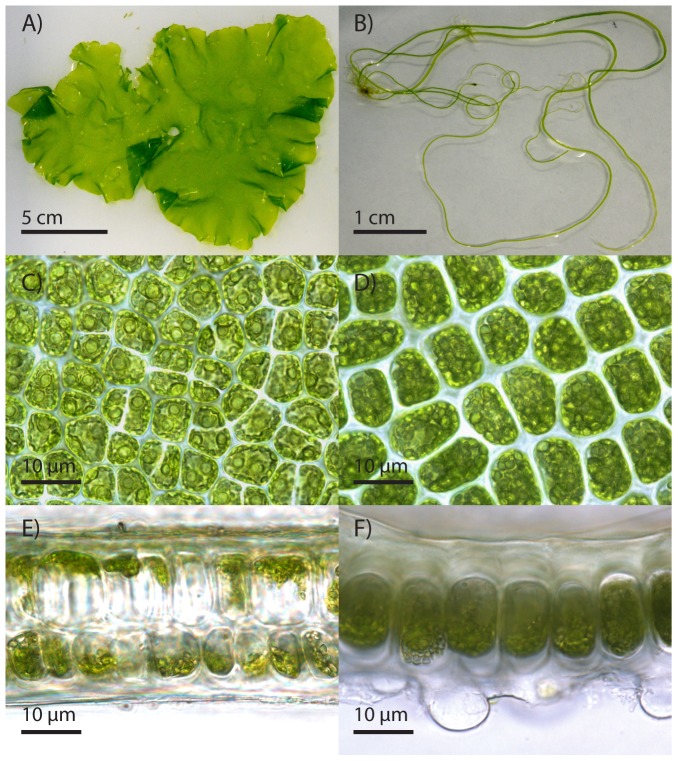
*U. ohnoi* and *U. sp. 3* light micrographs. Light micrographs of *Ulva ohnoi* and *U. sp. 3* in culture. Thallus morphology of *U. ohnoi* (A) and *U. sp. 3* (B); surface view of middle part of *U. ohnoi* thallus (C) and *U. sp. 3* thallus (D); transverse section of middle part of *U. ohnoi* thallus (E) and of a mature axis of *U. sp. 3* (F).

In total, five species of *Ulva* were found in Sydney, four in Brisbane and two in Townsville (not including the five samples where it was not possible to identify to species level). The most common blade species was *U. ohnoi* (14 strains, Fig. 3a) and this was also the only species found in all three locations. The most common filamentous species was *U. sp. 3* (9 strains, Fig. 3b) and this was only found in Brisbane and Townsville. The species found at aquaculture farms were *U. ohnoi, U. fasciata* and *U. sp. 3*.

### Growth Trials

**Figure 4:**
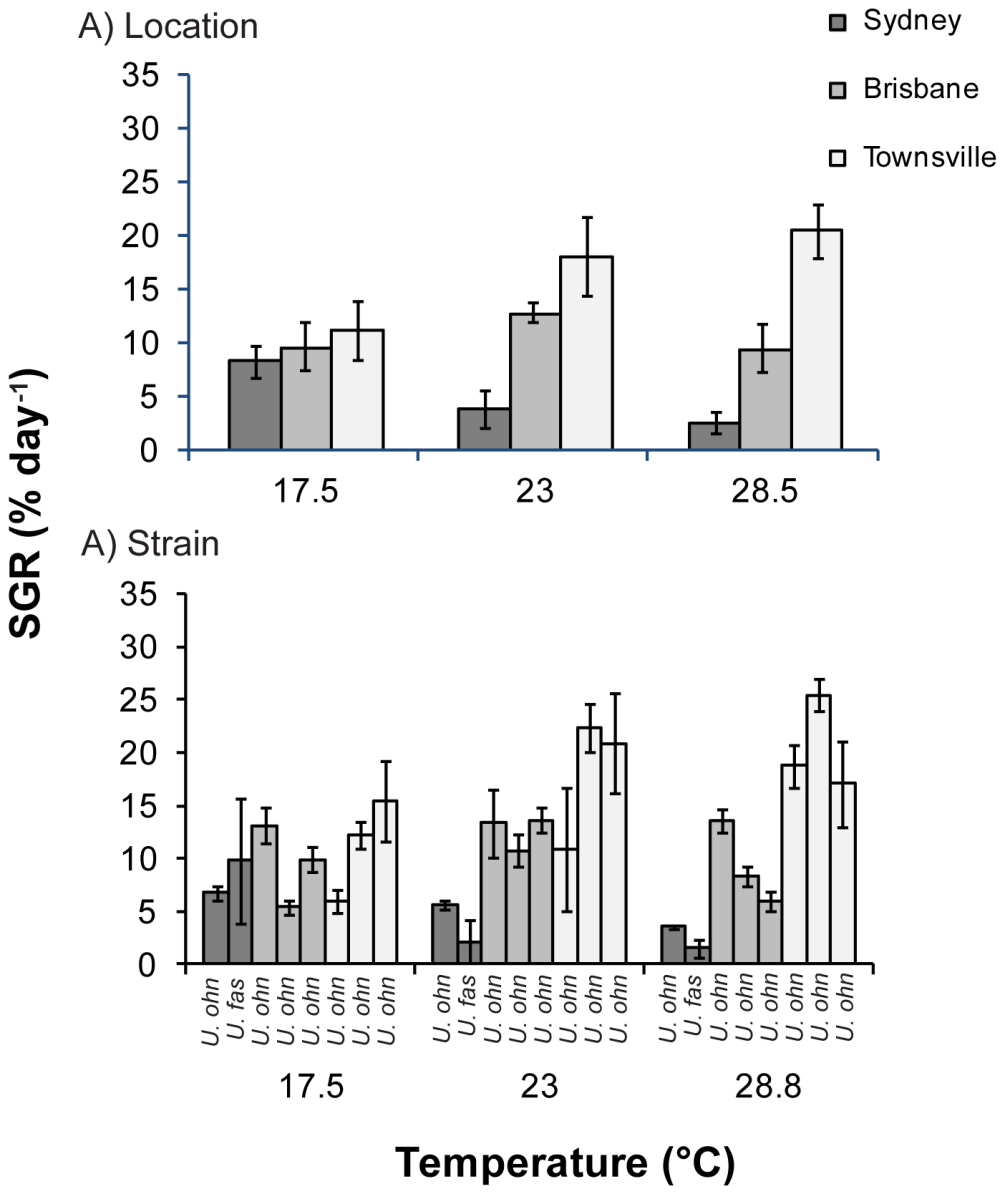
Specific growth rates of *Ulva* blades. Mean (±S.E.) specific growth rates (% day^−1^) of *Ulva* blades grown under three temperature treatments grouped by (A) collection location (Sydney, Brisbane and Townsville) and (B) *s*train. Strain codes from left to right are MA1, MR5, BI9, GC1, RC1, GFB1, GFB5 and JCU1. Species abbreviations: *U. fas - U. fasciata; U. ohn - U. ohnoi.*

Average specific growth rates of blade morphologies (predominantly *U. ohnoi*) ranged from 9.8% day^−1^ (±1.3 S.E.) at 17.5°C to 12.5% day^−1^ (±2.4 S.E.) at 25°C, while those of filamentous morphologies ranged from 15.6% day^−1^ (±2.9 S.E.) at 25°C to 18.4% day^−1^ (±2.3 S.E.) at 28.5°C. There was a significant difference in growth rates between blade morphology strains collected from different locations (Fig. 4a; Table 2). Across all temperature treatments, Townsville strains had the highest growth rates (11.2 (±2.8 S.E.) –20.4 (±2.6 S.E) % day^−1^) and Sydney strains had the lowest growth rates (2.5 (±1.0 S.E)−8.3 (±1.5 S.E.) % day^−1^) (Fig. 4a). For species from each location, the highest growth rates were attained at the temperature treatment that most closely matched ambient temperatures at that collection location. For example, Sydney strains grew most at 17.5°C (8.3 (±1.5 S.E.) % day^−1^), Brisbane strains at 23°C (12.6 (±0.9 S.E.) % day^−1^) and Townsville strains at 28.5°C (20.4 (±2.6 S.E) % day^−1^) (Fig. 4a).

**Table 2:** Results of permutational analyses of variance (PERMANOVAs) testing the effects of temperature (Te) and location (Lo) on specific growth rate of blade and filamentous *Ulva;* and the effects of temperature (Te) and strain (St) on specific growth rate of *Ulva ohnoi*.

		Blade *Ulva*		Filamentous *Ulva*		*U. ohnoi*	
Source	df	F	P	F	P	F	P
Te	2	0.52	0.635	2.25	0.073	0.644	0.607
Lo/St	2	**10.48**	**0.001**	2.57	0.068	**3.29**	**0.001**
Te × Lo/Te × St	4	0.88	0.482	**3.27**	**0.008**	1.28	0.178

Analyses were conducted in Primer v6 (Primer-E Ltd, UK) using Bray-Curtis dissimilarities on fourth root transformed data and 999 unrestricted permutations of raw data. Pseudo F (F) and P values are presented, significant terms shown in bold.

**Figure 5:**
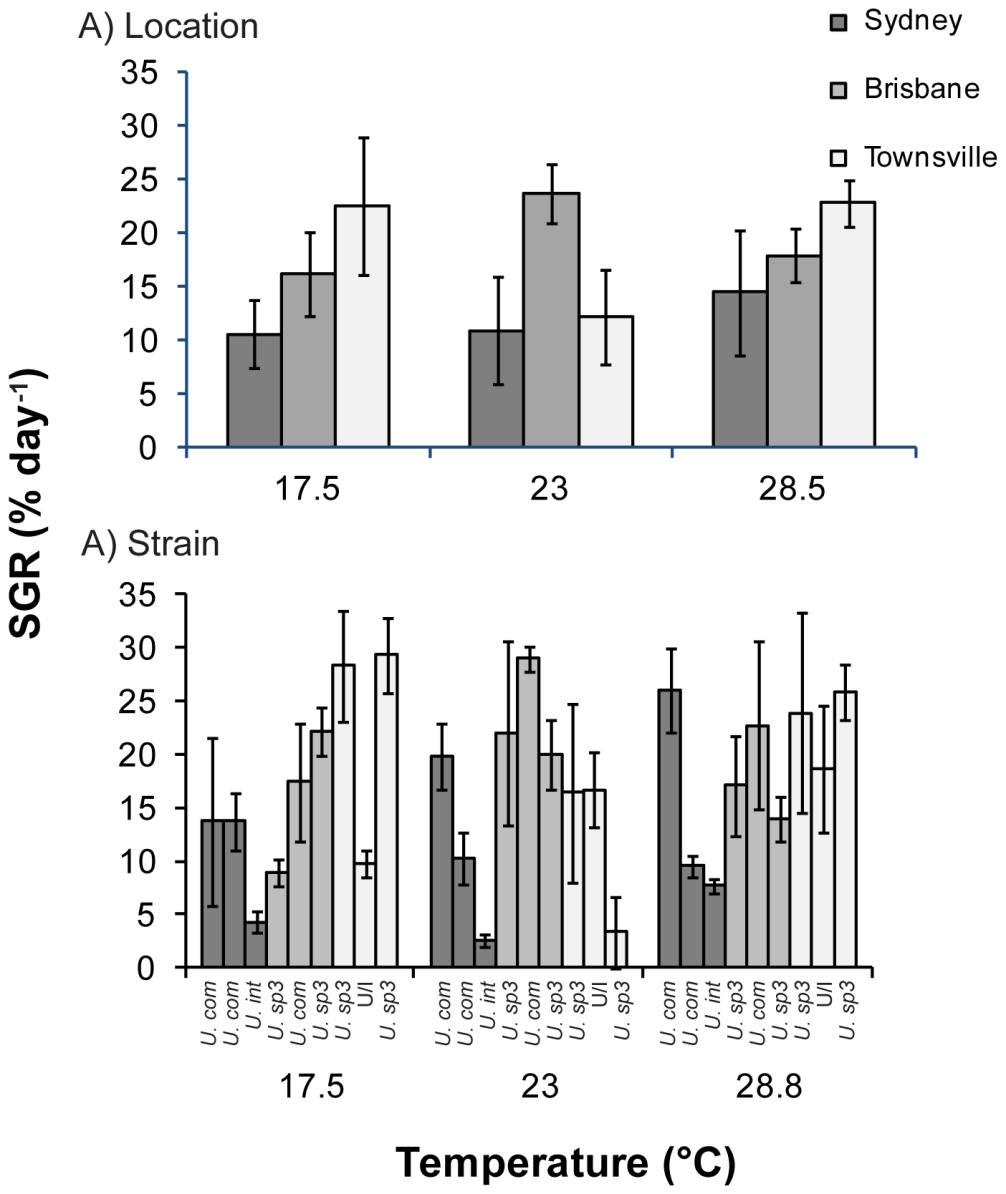
Specific growth rates of *Ulva* filaments. Mean (±S.E.) specific growth rates (% day^−1^) of *Ulva* filaments grown under three temperature treatments grouped by (A) collection location (Sydney, Brisbane and Townsville) and (B) *s*train. Strain codes from left to right are CL9, CL10, MR4, BI6, RC3, SB12, GFB2, JCU2 and PR3. Species abbreviations: *U. com - U.compressa; U. int - U.intestinalis; U. sp3 - U. sp. 3;* U/I *–* unidentified species.

Strains with filamentous morphologies had higher growth rates overall compared to blade morphologies; however, patterns were more variable, reflected by a significant temperature by location interaction effect (Fig. 5a and 5b; Table 2). Brisbane strains had the highest growth rates at 23°C (23.7 (±2.7 S.E.) % day^−1^), while Townsville strains had the highest growth rates at both 17.5 and 28.5°C (22.5 (±6.4 S.E.) and 22.8 (±2.2 S.E.) % day^−1^ respectively), Sydney strains had the lowest growth rates (10.6 (±3.1 S.E) –14.4 (±5.8 S.E.) % day^−1^) across all temperature treatments (Fig. 5a). One cause of the higher variation in growth rates between filamentous strains compared to blade strains may have been the occurrence of reproductive events in some of the filamentous strains. Over one third of all filamentous strains released propagules during the course of the experiment, possibly affecting growth and confounding the effects of temperature. There was no effect of location or temperature on propagule release.

When specifically analysing *U. ohnoi* growth rates to identify differences between strains within a single species, we found significant variation in growth between individual strains (Fig. 4b; Table 2). At each temperature treatment there was at least a two-fold difference in growth rates between samples collected from the same location. For example at 17.5°C one Townsville sample grew at 5.9% day^−1^ (±1.1 S.E) while another grew at 15.4% day^−1^ (±3.7 S.E), at 23°C one Townsville sample grew at 10.9% day^−1^ (±5.8 S.E) while another grew at 22.4% day^−1^ (±2.3 S.E), and at 28.5°C one Brisbane sample grew at 5.9% day^−1^ (±0.9 S.E) while another grew at 13.5% day^−1^ (±1.1 S.E) (Fig. 4b).

## Discussion

Our survey of natural populations and land-based aquaculture facilities in Eastern Australia identified six *Ulva* species, with *U. ohnoi* being the most common blade species and *U. sp.* 3 being the most common filamentous species. Both species occurred commonly at land-based aquaculture facilities in Townsville and Brisbane. Multiple strains of each species grew well in culture, surviving for more than 3 months under laboratory conditions. Specific growth rates of both blade and filamentous *Ulva* species were high (over 9% and 15% day^−1^ respectively) across a range of temperature treatments and comparable to, or greater than, growth rates recorded for species of *Ulva* and other algae used for bioremediation in both laboratory and outdoor studies, e.g., *U. clathrata* [12% day^−1^, 49], *U. lactuca* [17.9% day^−1^, 50], *U. rigida* [13.8% day^−1^, 51], *Gracilaria lemaneiformis* [11% day^−1^; 52], *Laminaria saccharina* [9% day^−1^; 53] and *Porphyra linearis* [16% day^−1^; 14]. These characteristics confirm the suitability of *Ulva* for algal bioremediation of aquaculture facilities in Eastern Australia.

Most land-based aquaculture facilities in Eastern Australia supply their ponds using unfiltered seawater (with its associated biota) taken directly from coastal environments. As such, endemic algal species often occur naturally in these ponds and can form large blooms [9]. These species are ideal candidates to target for bioremediation activities as their natural occurrence in aquaculture ponds demonstrates that they can survive and grow under conditions typical of land-based aquaculture, which are often quite different to conditions in natural intertidal habitats [9]. Additionally, the risk of cultivated species being over grown and outcompeted by other naturally occurring algae (e.g., [12]) will be low if cultivated species already form a significant component of the naturally occurring biota. Five of the six aquaculture facilities we sampled had species of *Ulva* growing in their ponds –*U. ohnoi* and *U. sp 3* each occurred at four farms in Townsville and Brisbane, while *U. fasciata* was found at a single farm in Brisbane. Use of these locally occurring species will minimise the risk of cultivated algae escaping and impacting on native biodiversity. Furthermore, their broad distribution in intertidal habitats across multiple locations facilitates the translocation of these species between aquaculture facilities. In addition to its high survival rates in culture for long time periods and fast growth rates, the natural occurrence of *U. ohnoi* at multiple aquaculture farms makes it an ideal species to select and develop for algal bioremediation in Eastern Australia. *U. sp. 3* also has potential to be used for bioremediation due to its broad distribution and natural occurrence at aquaculture farms. However, there was no differentiation in growth rate over other filamentous species and therefore alternative filamentous species, such as *U. compressa*, may also be suitable targets for bioremediation.

*Ulva ohnoi* has only recently been described as a species [54] and is most commonly recorded in “green tide” algal blooms around Japan [54], [55]. The same traits considered to be nuisance characteristics in species forming “green tide” algal blooms - high growth rates, board environmental tolerance and ability to use multiple sources of nitrogen [56], [57] - are in fact desirable characteristics in target species for bioremediation [3], [7]. Consequently, the occurrence of *U. ohnoi* in green tide blooms supports a role in bioremediation. Further support for the suitability of *U. ohnoi* for bioremediation is provided by the high growth rates (12.8–23.6% day^−1^) and nitrogen extraction rates (4.2–13.9 mg N g DW^−1^ day^−1^) reported for this species when cultivated next to a coastal fish farm in Japan [58], high nitrogen extraction rates of up to 12.9 mg N g DW^−1^ day^−1^ in Australia [59] and high biomass productivities of up to 40 g DW m^2^ day^−1^ when receiving effluent water from a fish hatchery (L. Mata, unpublished data). These rates are among the highest of those reported for a range of algae commonly used for bioremediation of aquaculture facilities.

Following identification of target species, suitable strains for bioremediation also need to be identified, particularly if populations are adapted to local environmental conditions. Strains of *Ulva ohnoi* had higher growth rates in temperatures corresponding to local conditions. These results were not an instantaneous effect as all samples were held at 23°C for three months prior to the start of the experiment. Studies of local adaptation in macroalgal species are relatively rare. Over large spatial scales (e.g., ocean wide) there is evidence both for [19], [21] and against [25] local adaptation of macroalgae. Likewise, over small spatial scales results are mixed with evidence for local adaptation over 100 s of kilometres [26] and two out of three populations showing evidence of local adaptation across intertidal zones [20]. The superior performance of local strains under temperatures corresponding to local conditions in the current study implies that strains of *U. ohnoi* are adapted to local temperatures to some extent. However, across all temperature treatments Townsville strains of *U. ohnoi* had the highest growth rates and Sydney strains had the lowest growth rates. Similarly, superior performance of a single “strain” across multiple habitat types or conditions has been reported for the red alga *Ceramium tenuicorne* [26] and the aquatic plant *Potamogeton pectinatus* [23]. Accordingly, it should not be assumed that growth rates will be highest in strains sourced from local environments and there may be selective pressure in local environments for traits other than growth and temperature tolerance.

In addition to significant differences in growth rates over large spatial scales (e.g., between locations), we found significant differences in growth rates between strains of *U. ohnoi* collected from the same location. Variation in growth between strains could be due to genetic differences (e.g., different genotypes); alternatively, strains may have the same genotype but respond differently to temperature treatments (e.g., phenotypic plasticity) [60]. As genetic differences between populations are expected to increase with geographic distance, genotypic differences may be causing variation in growth between locations, while variation within locations may result from phenotypic plasticity. Such a pattern is typical of widespread terrestrial plants, which often show phenotypic plasticity as well as high levels of genetic variation and adaptation to local environmental conditions [61], [62]. In contrast, widespread aquatic plants commonly have limited genetic variation [63] and macroalgae often exhibit striking phenotypic plasticity across large spatial scales [64], [65]. It is not possible to determine whether genotypic differences or phenotypic plasticity are causing variability in growth rates based on the results of our experiments or the molecular analyses we conducted. Although 11 out of the 14 strains of *U. ohnoi* we analysed had identical ITS and *tufA* DNA sequences, these molecular markers are intended for species discrimination rather than identification of genotypic differences within species. Further analysis using appropriate molecular markers is required to determine whether there are genotypic differences between strains of *U. ohnoi*. Regardless, significant differences in growth rates between strains of *U. ohnoi* opens up the possibility of isolating a strain which is able to maintain high growth rates across a range of temperatures and can be used for bioremediation of aquaculture facilities in Eastern Australia. While the domestication of macroalgal species for bioremediation is still in its infancy, the current study demonstrates that prior to undertaking genetic improvement programmes to selectively breed for desirable traits (e.g., [66]), large gains in productivity and bioremediation capability can be achieved by utilising existing natural genetic variation to identify and isolate fast growing species and strains.

## Supporting Information

Table S1 **Sample identification and collection information.** List of samples, collection date and location, initial species identification based on phylogenetic trees constructed using ITS and *tufA* sequence data, and final species identification used in this study. See results section for more detail on the rationale for final species identification.
(DOCX)
