# Correction: Algal Bioremediation of Waste Waters from Land-Based Aquaculture Using *Ulva*: Selecting Target Species and Strains

**DOI:** 10.1371/journal.pone.0231281

**Published:** 2020-03-27

**Authors:** Rebecca J. Lawton, Leonardo Mata, Rocky de Nys, Nicholas A. Paul

There is an error in Fig 1. The sequence “Ulva australis (AF099726)” should be replaced with “Ulva lactuca (AF099725).” Please see the correct Fig 1 here.

**Fig 1:**
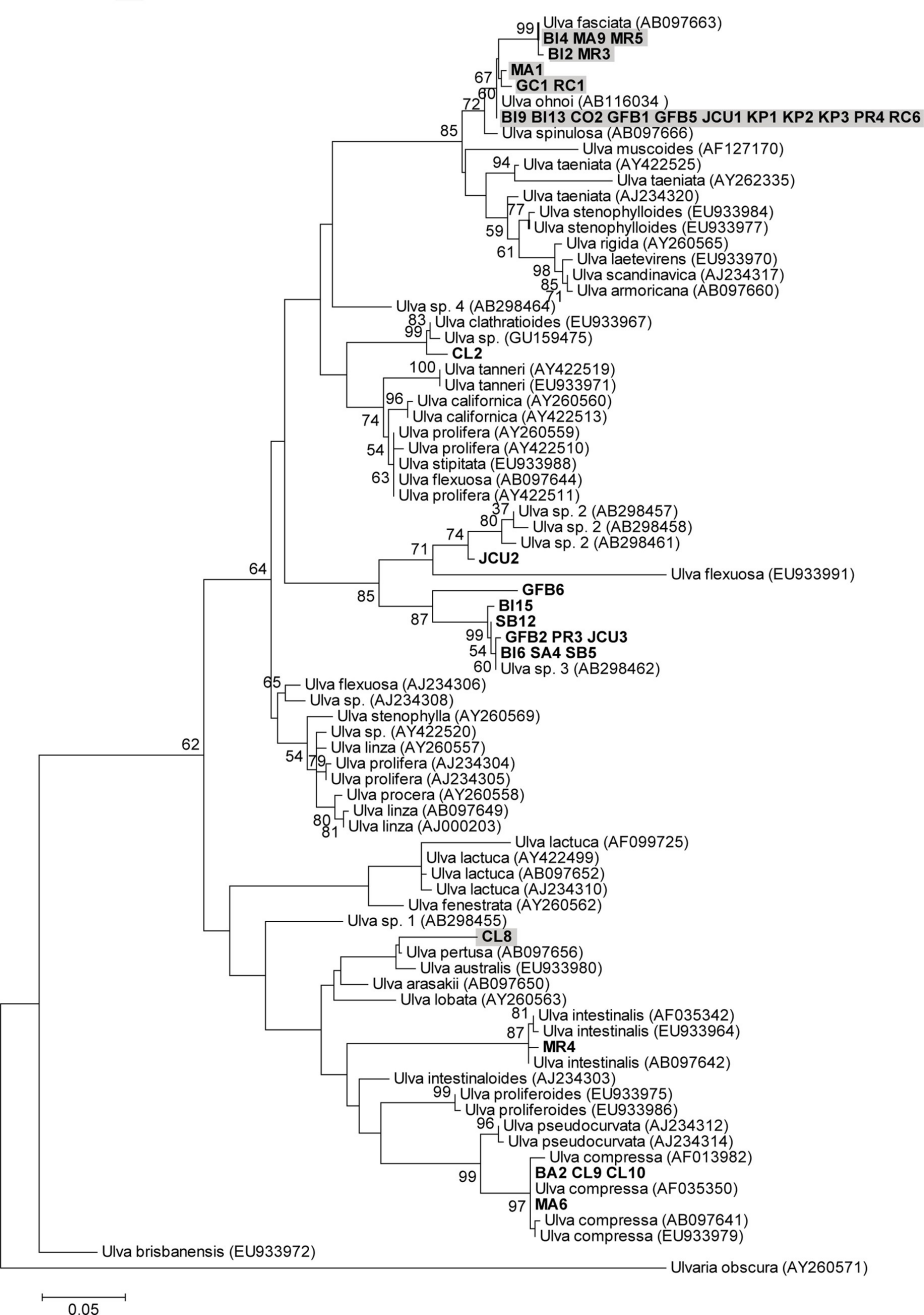
*Ulva* ITS phylogenetic tree. Maximum likelihood tree of *Ulva* internal transcribed spacer (ITS) sequence data (scale at bottom). Numbers near each node refer to bootstrap support values, nodes with <50% bootstrap support are not labelled. Samples collected in this study shown in bold. Shading indicates strains with blade morphologies. Numbers accompanying the species names are GenBank accession numbers for the sequences used in the analysis.
