# Supplementary material for: Algal Bioremediation of Waste Waters from Land-Based Aquaculture Using Ulva: Selecting Target Species and Strains
Source: PLoS One. 2013 Oct 15;8(10):e77344. doi: 10.1371/journal.pone.0077344 (PMC3797103; doi:10.1371/journal.pone.0077344)
Supplement: Table S1 — Sample identification and collection information. List of samples, collection date and location, initial species identification based on phylogenetic trees constructed using ITS and tufA sequence data, and final species identification used in this study. See results section for more detail on the rationale for final species identification. (DOCX) [file pone.0077344.s001.docx]

**Table S1 Sample identification and collection information**

List of samples, collection date and location, initial species identification based on phylogenetic trees constructed using ITS and *tufA* sequence data, and final species identification used in this study. See results section for more detail on the rationale for final species identification.

|  | **Collection details** | | **Initial species identification^1^** | | **Final identification** | | | |
| --- | --- | --- | --- | --- | --- | --- | --- | --- |
| **Strain** | **Date** | **Location** | **ITS** | ***tufA*** | | **Species^2^** | **Rationale** |  |
| BA2 | 7/11/12 | Bare Island, NSW  33°59'32" S, 151°13'54" E | *Ulva compressa* | *U. compressa* | | *U. compressa* | Identical to ITS and *tufA* sequences for *U. compressa* |  |
| BI2 | 24/10/12 | Bribie Island, QLD  27°03'14" S, 153°11'38" E | *U. fasciata* | *U. fasciata* | | *U. fasciata* | Identical to ITS and *tufA* sequences for *U. fasciata* |  |
| BI4 | 24/10/12 | Bribie Island, QLD  27°03'14" S, 153°11'38" E | *U. fasciata* | *U. ohnoi* | | U/I | *tufA* sequence identical to *U. ohnoi*, ITS sequence identical to *U. fasciata*, species identification not possible |  |
| BI6 | 24/10/12 | Bribie Island, QLD  27°03'14" S, 153°11'38" E | *U. sp. 3* | U/R | | *U. sp. 3* | Identical to ITS sequence for *U. sp. 3* from Shimada et al [48] |  |
| BI9 | 24/10/12 | Bribie Island, QLD  27°03'12" S, 153°11'38" E | *U. ohnoi* | *U. ohnoi* | | *U. ohnoi* | Identical to ITS and *tufA* sequences for *U. ohnoi* |  |
| BI13 | 24/10/12 | Bribie Island, QLD  27°03'14" S, 153°11'38" E | *U. ohnoi* | *U. ohnoi* | | *U. ohnoi* | Identical to ITS and *tufA* sequences for *U. ohnoi* |  |
| BI15 | 24/10/12 | Bribie Island, QLD  27°03'14" S, 153°11'40" E | *U. sp. 3* | N/C | | *U. sp. 3* | ITS sequence falls in same clade as *U. sp. 3* from Shimada et al (2008), *tufA* sequence did not match any Genbank samples |  |
| CL2 | 6/11/12 | Clovelly, NSW  33°54'55" S, 151°16'04" E | *U. clathratioides* | *U. torta* | | U/I | ITS sequence falls in the same clade as *U. clathratioides*, *tufA* sequences falls in the same clade as *U. torta*, species identification not possible |  |
| CL7 | 7/11/12 | Clovelly, NSW  33°59'33" S, 151°13'53" E | U/R | *U. fasciata* | | *U. fasciata* | Identical to *tufA* sequences for *U. fasciata* |  |
| CL8 | 7/11/12 | Clovelly, NSW  33°54'53" S, 151°16'15" E | *U. australis* | *U. australis* | | *U. australis* | Identical to *tufA* sequence for *U. australis* and ITS sequence falls in the same clade as *U. australis* |  |
| CL9 | 7/11/12 | Clovelly, NSW  33°54'53" S, 151°16'15" E | *U. compressa* | *U. compressa* | | *U. compressa* | Identical to ITS and *tufA* sequences for *U. compressa* |  |
| CL10 | 7/11/12 | Clovelly, NSW  33°54'53" S, 151°16'15" E | *U. compressa* | *U. compressa* | | *U. compressa* | Identical to ITS and *tufA* sequences for *U. compressa* |  |
| C02 | 7/11/12 | Coogee, NSW  33°55'11" S, 151°15'37" E | *U. ohnoi* | *U. ohnoi* | | *U. ohnoi* | Identical to ITS and *tufA* sequences for *U. ohnoi* |  |
| GC1 | 27/11/12 | Gold Coast Marine, QLD  27°42'26" S, 153°19'25" E | M/S | *U. ohnoi* | | *U. ohnoi* | Identical to *tufA* sequence for *U. ohnoi* and ITS sequence falls in the same clade as *U. ohnoi* and the closely related *U. fasciata* |  |
| GFB1 | 22/11/12 | Good Fortune Bay Fisheries, QLD  19°56'24" S, 147°55'45" E | *U. ohnoi* | *U. ohnoi* | | *U. ohnoi* | Identical to ITS and *tufA* sequences for *U. ohnoi* |  |
| GFB2 | 22/11/12 | Good Fortune Bay Fisheries, QLD  19°56'24" S, 147°55'45" E | *U. sp. 3* | U/R | | *U. sp. 3* | ITS sequence falls in same clade as *U. sp. 3* from Shimada et al [48] |  |
| GFB5 | 22/11/12 | Good Fortune Bay Fisheries, QLD  19°56'21" S, 147°55'59" E | *U. ohnoi* | *U. ohnoi* | | *U. ohnoi* | Identical to ITS and *tufA* sequences for *U. ohnoi* |  |
| GFB6 | 24/3/12 | Good Fortune Bay Fisheries, QLD  19°56'32" S, 147°55'50" E | N/C | N/C | | U/I | Does not form clades with any GenBank samples, species identification not possible |  |
| JCU1 | 22/11/12 | James Cook University, QLD  19°19'45" S, 146°45'41" E | *U. ohnoi* | *U. ohnoi* | | *U. ohnoi* | Identical to ITS and *tufA* sequences for *U. ohnoi* |  |
| JCU2 | 22/11/12 | James Cook University, QLD  19°19'41" S, 146°45'41" E | N/C | N/C | | U/I | Does not form clades with any GenBank samples, species identification not possible |  |
| JCU3 | 24/3/12 | James Cook University, QLD  19°19'57" S, 146°45'33" E | *U. sp. 3* | N/C | | *U. sp. 3* | ITS sequence falls in same clade as *U. sp. 3* from Shimada et al [48], *tufA* sequence did not match any Genbank samples |  |
| KP1 | 11/4/13 | Townsville, QLD  19°14'24" S, 146°47'58" E | *U. ohnoi* | *U. ohnoi* | | *U. ohnoi* | Identical to ITS and *tufA* sequences for *U. ohnoi* |  |
| KP2 | 11/4/13 | Townsville, QLD  19°14'26" S, 146°47'32" E | *U. ohnoi* | *U. ohnoi* | | *U. ohnoi* | Identical to ITS and *tufA* sequences for *U. ohnoi* |  |
| KP3 | 11/4/13 | Townsville, QLD  19°14'25" S, 146°47'43" E | *U. ohnoi* | *U. ohnoi* | | *U. ohnoi* | Identical to ITS and *tufA* sequences for *U. ohnoi* |  |
| MA1 | 7/11/12 | Malabar, NSW  33°58'05" S, 151°15'16" E | M/S | *U. ohnoi* | | *U. ohnoi* | Identical to *tufA* sequence for *U. ohnoi* and ITS sequence falls in the same clade as *U. ohnoi* and the closely related *U. fasciata* |  |
| MA6 | 7/11/12 | Malabar. NSW  33°58'07" S, 151°15'15" E | *U. compressa* | *U. compressa* | | *U. compressa* | Identical to ITS and *tufA* sequences for *U. compressa* |  |
| MA9 | 7/11/12 | Malabar, NSW  33°58'09" S, 151°15'20" E | *U. fasciata* | *U. fasciata* | | *U. fasciata* | Identical to ITS and *tufA* sequences for *U. fasciata* |  |
| MR3 | 7/11/12 | Maroubra, NSW  33°57'15" S, 151°15'33" E | *U. fasciata* | *U. fasciata* | | *U. fasciata* | Identical to ITS and *tufA* sequences for *U. fasciata* |  |
| MR4 | 7/11/12 | Maroubra, NSW  33°57'15" S, 151°15'34" E | *U. intestinalis* | *U. intestinalis* | | *U. intestinalis* | Identical to *tufA* sequence for *U. intestinalis* and ITS sequence falls in the same clade as *U. intestinalis* |  |
| MR5 | 7/11/12 | Maroubra, NSW  33°57'15" S, 151°15'35" E | *U. fasciata* | *U. fasciata* | | *U. fasciata* | Identical to ITS and *tufA* sequences for *U. fasciata* |  |
| PR3 | 22/11/12 | Pacific Reef Fisheries, QLD  19°28'45" S, 147°28'46" E | *U. sp. 3* | N/C | | *U. sp. 3* | ITS sequence falls in same clade as *U. sp. 3* from Shimada et al [48], *tufA* sequence did not match any Genbank samples |  |
| PR4 | 22/11/12 | Pacific Reef Fisheries, QLD  19°28'45" S, 147°28'46" E | *U. ohnoi* | *U. ohnoi* | | *U. ohnoi* | Identical to ITS and *tufA* sequences for *U. ohnoi* |  |
| RC1 | 24/10/12 | Redcliffe, QLD  27°15'24" S, 153°05'43" E | M/S | *U. ohnoi* | | *U. ohnoi* | Identical to *tufA* sequence for *U. ohnoi* and ITS sequence falls in the same clade as *U. ohnoi* and the closely related *U. fasciata* |  |
| RC3 | 24/10/12 | Redcliffe, QLD  27°15'26" S, 153°05'49" E | U/R | *U. compressa* | | *U. compressa* | Identical to *tufA* sequence for *U. compressa* |  |
| RC6 | 24/10/12 | Redcliffe, QLD  27°15'46" S, 153°06'18" E | *U. ohnoi* | *U. ohnoi* | | *U. ohnoi* | Identical to ITS and *tufA* sequences for *U. ohnoi* |  |
| SA4 | 6/12/12 | Australian Prawn Farms, QLD  21°42'51" S, 149°26'37" E | *U. sp. 3* | N/C | | *U. sp. 3* | Identical to ITS sequence for *U. sp. 3* from Shimada et al [48], *tufA* sequence did not match any Genbank samples |  |
| SB1 | 24/10/12 | Caloundra, QLD  26°47'25" S, 153°08'44" E | U/R | N/C | | U/I | *tufA* sequence does not form clades with any GenBank samples, species identification not possible |  |
| SB5 | 24/10/12 | Caloundra, QLD  26°47'24" S, 153°08'46" E | *U. sp. 3* | N/C | | *U. sp. 3* | Identical to ITS sequence for *U. sp. 3* from Shimada et al [48], *tufA* sequence did not match any Genbank samples |  |
| SB12 | 24/10/12 | Caloundra, QLD  26°47'24" S, 153°08'48" E | *U. sp. 3* | N/C | | *U. sp. 3* | ITS sequence falls in same clade as *U. sp. 3* from Shimada et al [48], *tufA* sequence did not match any Genbank samples |  |
| TV3 | 23/11/12 | Townsville, QLD  19°14'48" S, 146°48'45" E | U/R | N/C | | *U. sp. 3* | tufA sequence falls in same clade as other samples identified as *U. sp. 3* from Shimada et al [48] |  |

^1^ Abbreviations for sample outcome: U/R - unreadable sequence; N/C – formed a distinct clade that did not contain any published Genbank sequences; M/S – falls within a clade containing published Genbank sequences from multiple species

^2^ U/I - unidentifiable
